# ANN-Based Online Parameter Correction for PMSM Control Using Sphere Decoding Algorithm

**DOI:** 10.3390/s26020553

**Published:** 2026-01-14

**Authors:** Joseph O. Akinwumi, Yuan Gao, Xin Yuan, Sergio Vazquez, Harold S. Ruiz

**Affiliations:** 1 School of Engineering, University of Leicester, Leicester LE1 7RH, UK; joa21@leicester.ac.uk (J.O.A.); dr.harold.ruiz@leicester.ac.uk (H.S.R.); 2 Department of Electronic & Electrical Engineering, University of Strathclyde, Glasgow G1 1RX, UK; xin.yuan@strath.ac.uk; 3 Electronic Engineering Department, Universidad de Sevilla, 41004 Sevilla, Spain; sergi@us.es

**Keywords:** sphere decoding algorithm, permanent magnet synchronous motor, artificial neural network, model predictive control

## Abstract

This work addresses parameter mismatch in Permanent Magnet Synchronous Motor (PMSM) drives, focusing on performance degradation caused by variations in flux linkage and inductance arising under realistic operating uncertainties. An artificial neural network (ANN) is trained to estimate these parameter shifts and update the controller model online. The procedure comprises three steps: (i) data generation using Sphere Decoding Algorithm-based Model Predictive Control (SDA-MPC) across a mismatch range of ±50%; (ii) offline ANN training to map measured features to parameter estimates; and (iii) online ANN deployment to update model parameters within the SDA-MPC loop. MATLAB /Simulink simulations show that ANN-based compensation can improve current tracking and THD under many mismatch conditions, although in some cases—particularly when inductance is overestimated—THD may increase relative to nominal operation. When parameters return to nominal values the ANN adapts accordingly, steering the controller back toward baseline performance. The data-driven adaptation enhances robustness with modest computational overhead. Future work includes hardware-in-the-loop (HIL) testing and explicit experimental study of temperature-dependent effects.

## 1. Introduction

Model Predictive Control (MPC), as the name suggests, relies on an accurate system model to predict plant behavior [1]. The accuracy of this model is crucial, as incorrect predictions can significantly degrade system performance. However, system parameters often deviate due to manufacturing inconsistencies, aging, or temperature variations. For instance, an increase in ambient temperature by 100 °C can lead to a 20% reduction in flux [2]. Since offline parameter identification is time-consuming, several online approaches have been proposed. In [3], a Kalman filter and a Luenberger observer were used to estimate rotor positions. An adaptive sliding mode observer was employed to extract the third harmonic flux linkage [4], while in [5,6] a disturbance active rejection control based on a PID controller was introduced in induction motors to compensate for and predict parameter mismatch. Additionally, in [7], an extended state observer was proposed for estimating actual inductance using the permanent magnet synchronous machine (PMSM) incremental model. A comprehensive review of advanced control strategies for power converters is provided in [8].

These strategies can be broadly classified into model-based and data-driven approaches. The model-based methods include the techniques discussed earlier, while data-driven approaches leverage artificial intelligence (AI) techniques, such as artificial neural networks (ANNs), model free predictive control approaches and fuzzy control. Data-driven methods utilize input–output data to approximate system behaviour and optimize control strategies. Ref. [9] introduced an improved flux observer incorporating fuzzy algorithms to eliminate flux linkage disturbances. In [10], presents a fully model-free predictive current control strategy, where the PMSM dynamics are represented by ultralocal models and estimated using a fixed-time observer and extremum-seeking adaptation. This approach eliminates the need for prior model knowledge, in [11,12], represent two recent trends in MPC research: parameter-free reformulations and observer-based robust predictive control. The aforementioned strategies highlight a growing trend toward model-free control, where system dynamics are estimated or approximated without relying on detailed physical models. In this context, Artificial Neural Networks (ANNs) offer a powerful data-driven alternative. ANNs, particularly feedforward architectures, act as universal function approximators capable of learning complex nonlinear mappings directly from data, making them well-suited for predictive control tasks in systems such as PMSMs [13]. ANNs are categorized into three main types: feedforward, convolutional, and recurrent neural networks [14]. Among them, feedforward ANNs are the simplest and act as universal function approximators with strong generalization capabilities [15]. Consequently, this study primarily investigates the application of feedforward ANNs in power converters. Several studies have explored ANN applications in power electronics. For example, in [16], MPC was integrated with a feedforward ANN to minimize total harmonic distortion (THD) for both linear and nonlinear loads, thereby enhancing system performance. In [17], an ANN was utilized for real-time weighting factor tuning to regulate switching frequency independent of the operating point. Additionally, Ref. [18] proposed an ANN-based replacement for the Sphere Decoding Algorithm (SDA) in an RL load configuration, aiming to reduce computational complexity and online calculations. In [19], a novel AI-based inverse application method was introduced for power electronic converters, optimizing droop coefficients in microgrid applications and extending the operation range of modular multilevel converters under unbalanced grid conditions. In [20] an RNN-based robust adaptive model predictive speed control scheme was proposed for PMSM speed regulation under uncertain mechanical parameters, such as inertia and external load torque. The approach employed a discrete-time mechanical parameter observer (DTMPO) for online parameter identification, while the RNN-based algorithm solved the MPC optimisation problem in real time, thereby improving robustness against parameter mismatch. In [21], neural-network-based parameter estimation of induction motors was proposed, where both feedforward and recurrent neural networks were applied during speed control to estimate parameters such as rotor resistance and mutual inductance, significantly improving the performance of the control system. Unlike conventional AI-based approaches that replicate power system controllers, this work focuses on using ANNs to determine optimal control parameters, such as flux and inductance. In [22], a model-free approach based on ANN was investigated and had superior performance in terms of robustness and lower THD when compared to conventional MPC.

In [23], parameter mismatch misidentification led to very high total harmonic distortion (THD) in some cases, while in others the switching frequency increased to compensate for an inductance mismatch. With respect to flux mismatch, the impact was primarily observed on the reference currents when the mismatch was introduced at the controller level. Specifically, for certain flux values, the $i_{d}$ reference appeared shifted upward compared to the actual $i_{d}$ current, giving the impression of an undershoot, whereas for other values it was shifted downward, again resembling an undershoot. This effect occurred because the controller flux or inductance values were altered while keeping the machine parameters constant. In contrast, the present study investigates the opposite scenario, where the controller parameters (flux and inductance) are kept constant while the corresponding machine parameters are varied. The flux and inductance were chosen as key parameters to be varied because they have more influence on the steady state of the PMSM [24,25]. This distinction is crucial, as mismatch originating in the machine itself requires a different identification and compensation strategy compared to mismatch introduced at the controller level.

Although observer-based approaches (e.g., Kalman, Luenberger, or extended state observers) can provide accurate estimates, they typically rely on detailed models [26], are sensitive to measurement noise [27], and usually require more tuning work [28]. Similarly, model-free MPC techniques remove explicit model dependency [29], but require significant online calculation [30] and do not naturally separate the effects of flux and inductance mismatch. In contrast, an ANN-based estimator offers a data-driven, lightweight, and fast alternative that can exploit simple statistical features such as the mean and ripple of $i_{q}$ to decouple flux from inductance effects, while maintaining low runtime complexity. When trained on appropriately selected data, ANN can become a dedicated surrogate model for parameter identification. This makes ANN particularly attractive for real-time PMSM applications where robustness and efficiency are critical.

### 1.1. Recent Advances in ANN-Based PMSM Control

Recent studies have highlighted the growing role of ANNs in PMSM drives. Ref. [31] introduced a DPCC-VCMU scheme combining neural-network-based multi-parameter identification with voltage coefficient matrix update, enhancing robustness against model parameter mismatch. Ref. [32] developed a reinforcement learning neural network-assisted fractional-order sliding mode controller (RLNNA-FOSMC) for PMSM drives, enabling adaptive parameter tuning and reduced chattering. Ref. [33] proposed an ANN-assisted DSVM-MPC scheme where an offline-trained ANN replaces the vector selection process, achieving faster decision-making and lower computational complexity. Ref. [34] combined a simplified thermal model with a lightweight ANN for PMSM temperature estimation, demonstrating low-complexity, accurate online estimation. A multilayer perceptron (MLP)–based detector was developed for identifying and classifying current-sensor faults in PMSM drives without additional sensors or estimators [35]. This highlights the growing role of neural networks as virtual sensing tools in electric drives—a concept extended in this work to the online estimation of flux and inductance mismatches for adaptive control. Similarly, an ANN-based DTC algorithm for open-end winding induction motors replaced lookup tables and hysteresis controllers for optimal voltage selection [36].

### 1.2. Justification for SDA Selection

In [23], the Sphere Decoding Algorithm (SDA) was introduced as an efficient search method for long prediction horizon FCS-MPC of PMSMs. That study showed SDA offers an optimal balance between computational complexity and control accuracy, particularly for multi-step prediction horizons. Because the present study employs two- and three-step prediction for data generation, SDA is used as the branch-and-bound solver to ensure optimal cost-function minimization without exhaustive enumeration of all voltage-vector combinations. This guarantees high-quality, physically consistent input–output pairs for ANN training, providing accurate surrogate models for flux and inductance estimation.

### 1.3. Contribution of This Study

The main contributions of this paper are as follows:

- A data-generation framework using Long Prediction Horizon Finite Control Set MPC (LPH-FCS-MPC) under a wide range of parameter mismatch scenarios.
- A low-feature feedforward ANN estimator trained to capture flux and inductance mismatch effects using simple current-based features.
- Online integration of the ANN with the predictive controller to update parameters, reducing THD and preserving stability even under significant mismatch.
- Runtime profiling and analysis demonstrating the computational efficiency of the proposed ANN estimator.

The remainder of this paper is organized as follows: Section 2 presents the fundamental principles of feedforward artificial neural networks and their role in the proposed strategy, it also details the system model and formulates the FCS-MPC problem using the SDA-MPC. Section 3 outlines the ANN architecture and its application to online parameter identification. Section 4 outlines the ANN data collection and the ANN training performance. Section 5 discusses simulation results under different conditions, including variations in prediction horizons. Finally, Section 6 concludes the paper with key findings and suggestions for future work.

## 2. Feedforward ANN-Aided LPH FCS-MPC System Formulation

### 2.1. Feedforward Neural Networks

A Feedforward Artificial Neural Network (ANN), also referred to as a fully connected neural network, is among the earliest architectures developed in artificial intelligence [37]. Unlike conventional function-fitting methods such as polynomial or exponential regression, an Feedforward ANN does not require an explicitly defined functional form. Instead, the mapping between inputs and outputs is autonomously learned from data, enabling its application across a wide range of electrical engineering problems, including reliability-oriented design of power electronic converters [38,39] and power loss correction in permanent-magnet synchronous machines [40].

**Figure 1:**
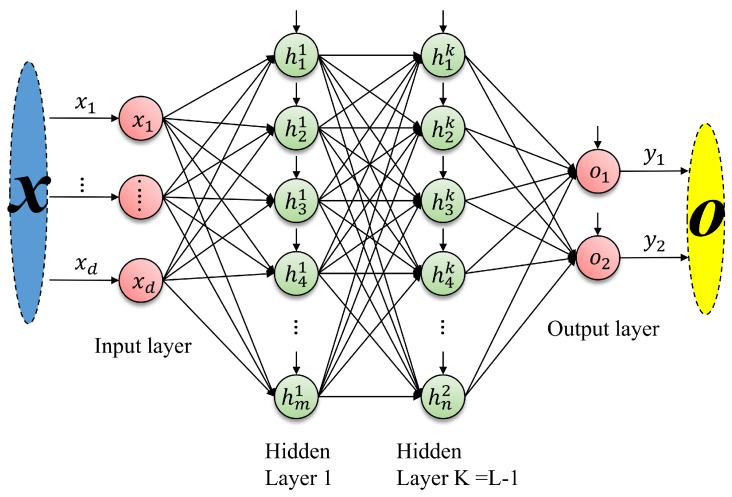
Diagram of a feedforward ANN.

A typical Feedforward ANN comprises an input layer, one or more hidden layers, and an output layer, as illustrated in Figure 1. The hidden layers perform nonlinear transformations that extract and separate relevant features from the input space [41]. Each neuron receives a weighted combination of signals from the previous layer, adds a bias term, and passes the result through an activation function. Formally, the output of neuron *i* in layer *l* is given by

(1) $$p_{i}^{l}=f_{\sigma }\left(\sum_{j=1}^{N_{l-1}}\omega_{ij}^{l}p_{j}^{l-1}+b_{i}^{l}\right),\;i=1,\ldots ,N_{l},$$

where $\omega_{ij}^{l}$ denotes the weight connecting neuron *j* in layer l−1 to neuron *i* in layer *l*, and $b_{i}^{l}$ is the corresponding bias. For hidden layers, the activation function $f_{\sigma }\left(\cdot \right)$ is commonly chosen as the sigmoid function:

(2) $$f_{\sigma }\left(A\right)=\frac{1}{1+e^{-A}},$$

while a linear activation is generally used at the output layer:

(3) $$o_{i}=\omega_{i}^{L}p_{i}^{L-1},\;i=1,\ldots ,N_{L}.$$

Through successive layers, the Feedforward ANN maps inputs $x_{i}$ ($i=1,\ldots ,N_{1}$) to outputs $o_{i}$, enabling complex nonlinear relationships to be approximated with arbitrary precision [15]. This universal approximation property, combined with its strong generalization ability, makes the feedforward ANN a suitable candidate for replacing complex physical models in predictive control and system identification tasks.

### 2.2. System Model and LPH-FCS-MPC Formulation

**Figure 2:**
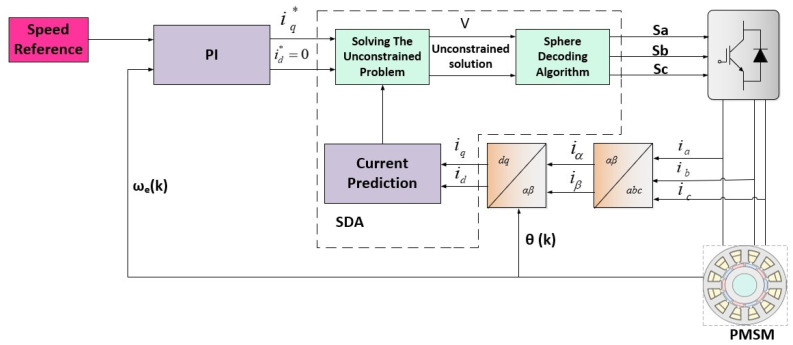
Illustration of sphere decoding algorithm for PMSM drive [23].

A two-level inverter with a PMSM drive is the system under consideration. The SDA-MPC framework is shown in Figure 2. The system variables are described in Table 1. The prediction model for the system is summarized in the same table.

The control input, $u_{k}$, represents the switching states of the power converter, where $u_{k}\in U$, the set of admissible switching states. The output, $y_{k}$, corresponds to the stator currents, as the primary control objective is current tracking. Since MPC is used, the state-space model is defined as

(4) $$x_{k+1}=Ax_{k}+Bu_{k}+\psi_{m}$$

(5) $$y_{k}=Cx_{k}$$

where the variables and matrices are defined in Table 1.

The following applies:

- $i_{d}$ and $i_{q}$ are the currents on the *d*- and *q*-axis, respectively;
- $R_{s}$ is the stator resistance;
- $L_{d}$ and $L_{q}$ are the *d*-axis and *q*-axis inductances;
- $\omega_{e}$ is the electrical angular speed of the rotor;
- $\psi_{m}$ is the permanent magnet flux linkage.

A key component of the MPC framework is the cost function, formulated as a two-norm expression:

(6) $$J={∥I_{dq}^{*}\left(k+N\right)-I_{dq}\left(k+N\right)∥}_{2}^{2}+\lambda {∥\Delta U∥}_{2}^{2}.$$

where $\Delta U=u_{k}-u_{k-1}$ denotes the change in switching state between consecutive samples (dimensionless).

This cost function consists of two main terms:

- **Tracking Error:** The squared norm of the difference between the reference current $I_{dq}^{*}$ and the predicted current $I_{dq}$. The algorithm selects $u_{k}$ that minimizes this error.
- **Control Effort:** This includes a weighting factor λ that balances switching frequency and THD. A higher λ reduces switching frequency but can increase THD.

As discussed in [42], multi-step prediction significantly improves performance in higher-order systems, especially in reducing harmonic distortion.

To extend the prediction to a horizon of length *N*, the control input sequence is defined as

$$U\left(k\right)={\left[\begin{array}{cccc} u_{k} & u_{k+1} & \ldots & u_{k+N-1} \end{array}\right]}^{T},$$

and the resulting SDA-based least-squares problem is expressed as [42]

(7) $$U_{\mathrm{opt}}\left(k\right)=\arg\min_{U(k)}{∥VU\left(k\right)-{\bar{U}}_{\mathrm{unc}}\left(k\right)∥}_{2}^{2},$$

where *V* is the lower-triangular system matrix mapping control increments to predicted states, and ${\bar{U}}_{\mathrm{unc}}\left(k\right)$ is the unconstrained input sequence from the model prediction. This formulation connects the predictive control problem to the Sphere Decoding Algorithm, which efficiently explores the discrete control set.

Since the predictive model relies on accurate machine parameters, any mismatch (e.g., inductance, flux linkage) degrades the cost evaluation. To address this, a feedforward Artificial neural network is employed to estimate the effective parameters online. The ANN outputs are used to update the model matrices *A*, *B* and ψ in the predictive formulation, thereby improving the accuracy of the SDA-based optimization.

### 2.3. Performance Metrics

#### 2.3.1. THD

In this work, THD is computed on the phase current signals of the PMSM, as these directly reflect the quality of the motor drive currents and are most relevant for torque ripple and machine losses. For completeness, the *q*-axis current is also monitored in the discussion, but all reported THD values correspond to the three-phase stator currents.

The computation follows the classical definition of THD as the ratio between the RMS value of all harmonic components and the RMS of the fundamental. For a generic current signal x(t), this can be expressed as [23]

(8) x(t)=Acos(ωt−ϕ)+(higherharmonics),

with the fundamental amplitude obtained from the orthogonal coefficients

(9) $$a_{cs}=\frac{2}{T}\int_{0}^{T}x\left(t\right)\cos\left(\omega t\right)\;dt,\;b_{sn}=\frac{2}{T}\int_{0}^{T}x\left(t\right)\sin\left(\omega t\right)\;dt,$$

(10) $$A=\sqrt{a_{cs}^{2}+b_{sn}^{2}}.$$

The RMS of the signal is

(11) $$\mathrm{RMS}=\sqrt{\frac{1}{T}\int_{0}^{T}x{\left(t\right)}^{2}\;dt},$$

and thus THD is defined as

(12) $$I_{\mathrm{THD}}=\sqrt{{\left(\frac{\mathrm{RMS}}{A}\right)}^{2}-1}.$$

In practice, this formulation is implemented in MATLAB R2023a/Simulink using FFT-based analysis over steady-state current waveforms, where the fundamental component is taken at the operating electrical frequency of the PMSM. This ensures reproducibility and allows direct comparison across operating conditions.

#### 2.3.2. Switching Frequency

The switching frequency $f_{sw}$ is calculated directly from the inverter switching states generated by the predictive controller. It is defined as the average number of device commutations per unit time, given by [43]

(13) $$f_{sw}=\lim_{M\to \infty }\frac{1}{m_{ck}\;M\;T_{s}}\sum_{k=0}^{M-1}{∥\Delta u\left(k\right)∥}_{1},$$

where Δu(k) is the change in the switching vector at time step *k*, $T_{s}$ is the sampling period, and $m_{ck}$ is the number of semiconductor devices per converter leg. For a two-level VSI, the correction factor is $c_{k}=2$ as in [43]. This metric provides a consistent measure of the effective converter operating frequency and switching effort across different scenarios.

#### 2.3.3. Stability Analysis

Ensuring closed-loop stability is a fundamental requirement for the predictive control of permanent magnet synchronous machines (PMSMs), particularly when adaptive mechanisms such as artificial neural networks (ANNs) are incorporated to estimate online parameter mismatches. While the proposed ANN-assisted Sphere Decoding Algorithm Model Predictive Control (ANN–SDA–MPC) enhances robustness and tracking performance [42], it is essential to verify that the control law guarantees stable current dynamics under parameter variations. To evaluate stability, a discrete-time Lyapunov-based criterion was employed. The Lyapunov candidate function was defined using the *d*–*q* current tracking errors as

(14) $$V\left(k\right)=\frac{1}{2}\left(e_{d}^{2}\left(k\right)+e_{q}^{2}\left(k\right)\right),$$

where $e_{d}\left(k\right)=i_{d,ref}\left(k\right)-i_{d}\left(k\right)$ and $e_{q}\left(k\right)=i_{q,ref}\left(k\right)-i_{q}\left(k\right)$ represent the current tracking errors in the *d* and *q* axes, respectively. For the closed-loop system to be stable, the Lyapunov function must remain positive definite, i.e.,

(15) V(k)>0,

and its discrete-time difference, defined as

(16) ΔV(k)=V(k+1)−V(k),

should be negative or non-positive on average:

(17) ΔV(k)<0onaverage.

In practice, ΔV(k) may oscillate between positive and negative values due to inverter switching actions and sampling effects inherent in finite control set predictive control (FCS–MPC). However, as long as the average value of ΔV(k) remains negative, the closed-loop system dissipates energy over time, satisfying Lyapunov stability in the mean sense.

The Lyapunov function and its difference were computed in real time during simulation to monitor system energy evolution. A decreasing trend in V(k) and a negative mean of ΔV(k) confirm that the proposed ANN–SDA–MPC maintains closed-loop stability under the considered parameter mismatches.

## 3. Proposed ANN Method

### 3.1. Artificial Neural Network Design for Parameter Mismatch Estimation

**Figure 3:**
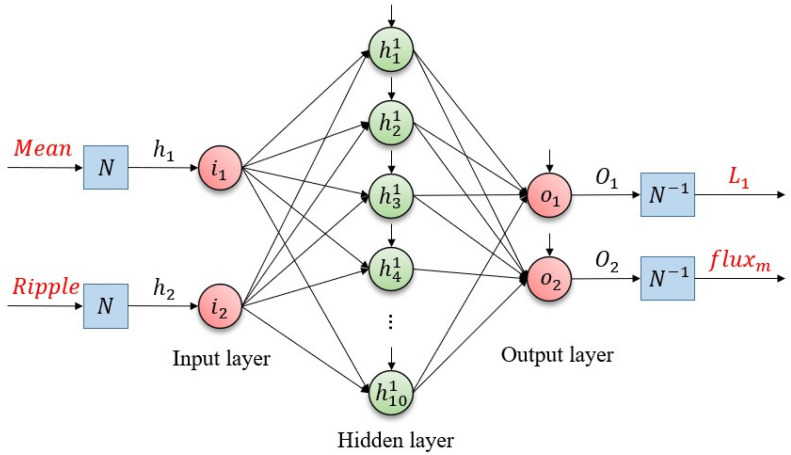
The proposed ANN design for parameter mismatch problem.

The neural network used in this study was designed using MATLAB’s Neural Network Fitting Tool (‘nftool’). As shown in Figure 3, a two-layer feedforward neural network architecture was employed, comprising

- Input layer: 2 neurons corresponding to the extracted features—mean ($x_{1}$) and ripple magnitude ($x_{2}$) of the $i_{q}$ current.
- Hidden layer: 10 neurons with sigmoid activation functions (tansig in MATLAB).
- Output layer: 2 neurons representing the estimated machine parameters—flux linkage ($y_{1}$) and inductance ($y_{2}$), with linear activation functions (purelin in MATLAB).

**Figure 4:**
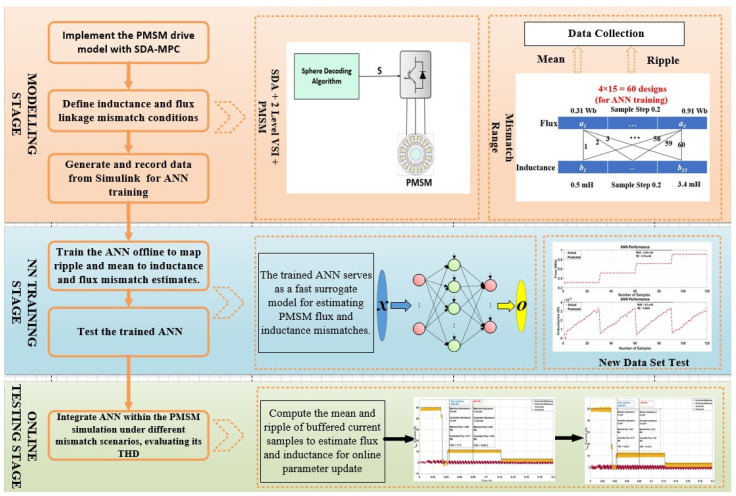
Overview of the proposed three-stage workflow for ANN-based parameter mismatch compensation in PMSM drives: (1) Modelling and Data Generation—PMSM with SDA-MPC simulated under ±50% flux and inductance variations; (2) Training Stage—offline ANN training and validation using simulated current and voltage data; (3) Online Testing—deployment of the trained ANN for real-time parameter update and performance evaluation under mismatch conditions.

The overall workflow of the proposed ANN-assisted SDA-MPC framework is illustrated in Figure 4, highlighting the modelling, training, and online testing stages used for parameter mismatch compensation.

### 3.2. Rationale for Feature Selection

The use of the mean and ripple of the $i_{q}$ current as ANN inputs is motivated by the distinct physical signatures of flux and inductance mismatches in the PMSM. Flux linkage mismatch primarily shifts the steady-state reference trajectory of the $i_{q}$ current, leading to systematic bias in its mean value. In contrast, inductance mismatch alters the current dynamics under a fixed sampling interval and switching frequency, directly manifesting as variations in the ripple magnitude. By jointly considering the mean and ripple, the proposed ANN is able to decouple flux and inductance effects with only two scalar features, ensuring both computational efficiency and robustness.

## 4. ANN Data Collection and Training Performance

### 4.1. Data Generation for ANN Training

The training dataset was obtained by systematically varying PMSM parameters while freezing all controller settings at their nominal values. Flux linkage (ψ) and stator inductance (*L*) were perturbed within ±50% of their nominal values, consistent with the problem statement. Step sizes of 10% were applied across this range, yielding a structured grid of design points. Each simulation was performed under constant mechanical speed (2500 r/min) and constant torque load, ensuring that observed variations in $i_{q}$ were solely attributable to parameter mismatches. The current sampling frequency was set to 100 kHz, and feature extraction was performed within the time window t=0.042–0.07 s of the total 0.2 s simulation period. Within this interval, the *q*-axis current ($i_{q}$) was sampled to compute two statistical descriptors: the mean value and the ripple magnitude. This window was deliberately selected after the initial transient response (t<0.042 s) ensuring that the extracted features represent stable system behavior dominated by parameter mismatch effects rather than dynamic load or speed transients. These mean and ripple values were then used as ANN inputs, allowing the network to learn a clean and physically consistent mapping between observable current characteristics and the underlying flux linkage and inductance parameters.

### 4.2. Training Procedure

The dataset was partitioned into 60 training cases and 120 testing cases, with a fixed random seed to ensure reproducibility. The ANN was trained using the Levenberg–Marquardt backpropagation algorithm (trainlm) in MATLAB, with the mean squared error (MSE) loss function. The training was conducted over a maximum of 200 epochs with early stopping enabled (validation patience of 10 epochs). Weights were initialized using MATLAB’s default Nguyen–Widrow scheme. No data augmentation was applied.

**Figure 5:**
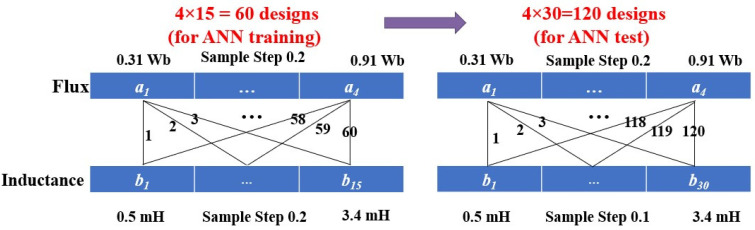
Data Collection Approach for ANN Training.

Two datasets were generated from systematic parameter variations of the PMSM. In the first dataset, the flux linkage ψ was varied from 0.31 to 0.91 Wb in steps of 0.2, while the *q*-axis inductance $L_{q}$ was varied from 0.5 mH to 3.4 mH in steps of 0.2 mH, yielding 60 samples as shown in Figure 5. This dataset was randomly partitioned into 70% training, 15% validation, and 15% testing subsets. To evaluate the generalization ability of the ANN, a second dataset was generated with the same flux variation but a finer inductance resolution (step size 0.1 mH), resulting in 120 samples. This second dataset was used exclusively for robustness testing. The network was trained in MATLAB using the Levenberg–Marquardt backpropagation algorithm (trainlm) with the mean squared error (MSE) as the loss function. Building on the extracted current features described in the previous subsection, the ANN was trained following an Inverse Application of Artificial Intelligence (IAAI) paradigm. Inverse Application of Artificial Intelligence (IAAI): Unlike conventional ANN training, where machine parameters are used as inputs to predict resulting current features, the ANN in this work is trained in an inverse manner. The mean and ripple of the $i_{q}$ current serve as inputs, while the corresponding flux linkage ψ and inductance $L_{q}$ serve as outputs. This IAAI approach allows the network to learn the mapping from observable electrical features back to underlying machine parameters, enabling real-time estimation of parameter mismatches directly from measured currents.

### 4.3. Performance Evaluation Metrics

To assess the accuracy of the proposed ANN model in estimating machine parameters, two error metrics were employed: the Mean Absolute Error (MAE) and the Relative Error (RE). These metrics quantify the deviation between the predicted outputs $Y_{\mathrm{pred}}$ and the true target values *Y*, thereby providing complementary insights into model performance.

The MAE is defined as

(18) $$\mathrm{MAE}=\frac{1}{N}\sum_{k=1}^{N}\left|Y\left(k\right)-Y_{\mathrm{pred}}\left(k\right)\right|,$$

where *N* denotes the total number of samples. This metric measures the average magnitude of absolute errors, offering an intuitive interpretation of the prediction accuracy in the same units as the target variables.

**Figure 6:**
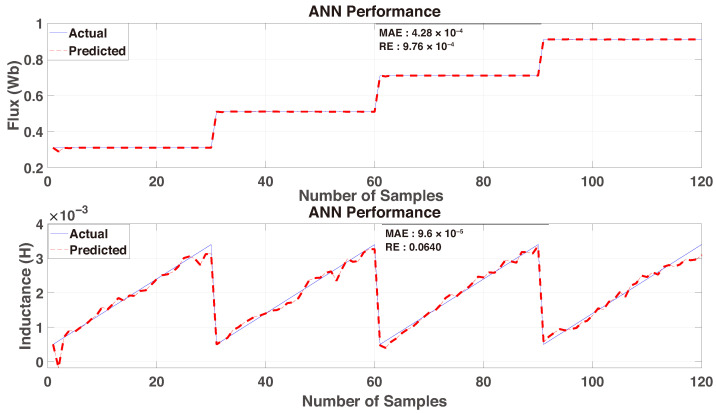
ANN test performance using the new dataset.

The RE, on the other hand, normalizes the prediction error with respect to the actual value, thereby expressing the deviation as a fraction of the true magnitude:

(19) $$\mathrm{RE}=\frac{1}{N}\sum_{k=1}^{N}\frac{\left|Y\left(k\right)-Y_{\mathrm{pred}}\left(k\right)\right|}{Y(k)+\epsilon },$$

where ϵ, $\epsilon =2.2204\times 10^{-16}$ is a small positive constant introduced to prevent division by zero. The RE provides a scale-invariant measure, particularly useful when target values vary over different ranges. Both metrics were computed independently for each estimated parameter (e.g., inductance and flux), enabling a clear evaluation of the ANN’s predictive performance across multiple outputs. While MAE emphasizes the absolute magnitude of prediction errors, RE highlights their proportional significance relative to the true values, offering a more comprehensive assessment. The top plot shown in Figure 6 corresponds to the flux prediction, where the MAE is $4.28\times 10^{-4}$, and the relative error is approximately $9.76\times 10^{-4}$. The bottom plot Figure 6 shows the inductance prediction, which yields an MAE of $9.6\times 10^{-5}$ and a relative error of 0.0640. While the network demonstrates strong tracking performance for flux linkage across a wide range of operating conditions, the inductance estimation shows moderate error levels, particularly in regions with rapid transitions. Nonetheless, the results support the ANN’s suitability for integration into adaptive control strategies, especially in scenarios where coarse parameter correction is sufficient for maintaining system performance.

### 4.4. ANN Runtime

**Figure 7:**
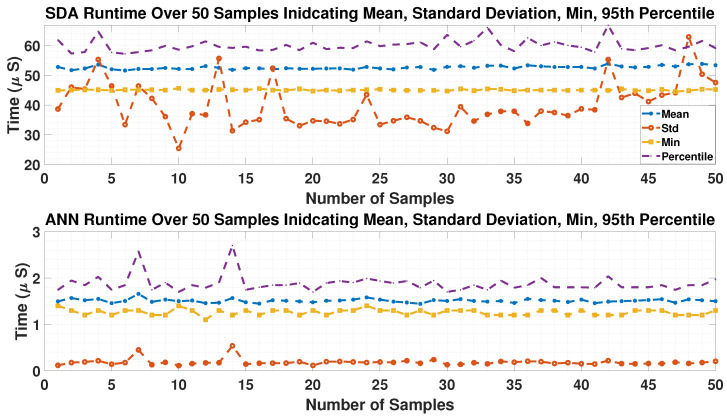
Execution time of the SDA-MPC and ANN.

To account for stochastic variability in runtime measurement, each simulation was repeated for 50 independent runs, with each run producing 20,000 timing samples. From each run, summary statistics (mean, standard deviation, minimum, and 95th-percentile execution time) were extracted for both the SDA-MPC and ANN modules. Since runtime is influenced by transient dynamics and solver branching depth, single-run statistics may not fully capture the overall computational profile. Therefore, Figure 7 illustrates the distribution of these summary statistics across the 50 runs, highlighting the run-to-run variation in each metric. The values reported in Table 2 correspond to the mean across all runs, with the standard deviation across runs provided in parentheses. This approach mitigates outlier effects and yields stable, statistically representative estimates of computational performance. Together with the raw per-cycle timing profile in Figure 7, these results confirm that while the SDA-MPC runtime exhibits variability, the ANN module remains lightweight and highly consistent.

These results complement the runtime distribution shown in Figure 7.

As seen in Table 2, the SDA-MPC exhibits an average execution time on the order of tens of microseconds, with relatively large variability (standard deviation of 39.78 μs) and a 95th-percentile close to 60 μs. This reflects the inherent dependence of SDA-MPC runtime on instantaneous system dynamics and search tree complexity. By contrast, the ANN estimator consistently executes in under 2 μs, with minimal variation across runs, confirming its negligible computational overhead. The combined analysis of Figure 7 and Table 2 demonstrates that while the SDA-MPC runtime distribution is broader, it remains within feasible limits for real-time PMSM control, and the ANN module adds virtually no additional computational burden.

## 5. Results

**Figure 8:**
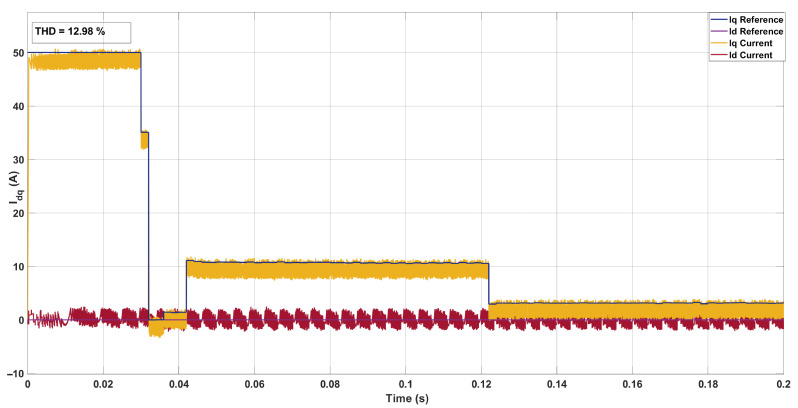
Current in the *dq*-axis for $T_{s}=$ 10 μs with Weighting factor ($\lambda_{u}=10$) for a three-step SDA-MPC.

The simulations were conducted in MATLAB/Simulink R2023a on a workstation equipped with an Intel(R) Core(TM) i9-10900 processor (3.70 GHz), 64 GB RAM, running Windows 10. The ANN configuration and training procedure are detailed in Section 3. In the simulation, the ANN was integrated into the SDA-MPC control loop to provide online estimates of flux linkage and inductance. Input features were collected within a 0.042–0.07 s time window and processed via a buffer to compute the mean and ripple of $i_{q}$. The ANN was activated at 0.08 s to generate parameter estimates, which were fed back to the controller in real time for the next control interval. To evaluate performance, parameter mismatches were introduced in the PMSM while keeping the controller parameters constant. The proposed method was tested under two different operating conditions to assess current tracking and THD. Each simulation lasted approximately 0.2 s of real time, with THD computed offline using the logged current waveforms.This configuration ensures full reproducibility of the simulation results, including ANN estimation, SDA-MPC integration, and the evaluation of flux and inductance mismatch compensation. Figure 8 shows the *dq* current for the PMSM in the absence of parameter mismatch.

### 5.1. System Configuration 1

**Figure 9:**
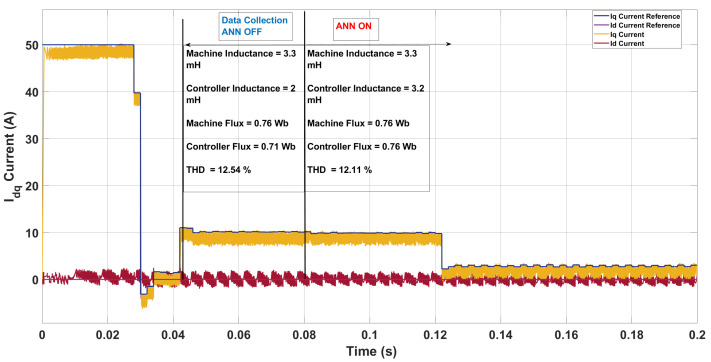
Current in the *dq*-axis for $T_{s}=$ 10 μs with $\lambda_{u}=10$ for a three-step SDA-MPC with mismatch of L = 3.3 mH and flux = 0.76 Wb.

In the baseline case with nominal parameters, the three-step SDA-MPC produced a total harmonic distortion (THD) of 13.34% in the phase current, with an average switching frequency of 5.8 kHz. In the first mismatch scenario (Figure 9), the machine inductance was increased to 3.3 mH and the actual flux linkage was 0.76 Wb, while the controller continued to operate with nominal settings of 2 mH and 0.71 Wb. The ANN was enabled at t=0.08 s after collecting training features between 0.042–0.07 s. Before compensation, the mismatch primarily in inductance caused the controller to apply less aggressive voltage transitions, which naturally filtered high-frequency current ripple. As a result, the phase-current THD decreased to 12.54%, albeit with a lower switching frequency of 4.03 kHz compared to the nominal case. After activation, the ANN correctly estimated the flux linkage at 0.76 Wb and slightly underestimated the inductance (3.2 mH versus the true 3.3 mH). This partial correction increased the switching activity (to 4.44 kHz) and further smoothed the current trajectory, yielding a slightly reduced THD of 12.11%. These results illustrate that under certain mismatched conditions, a reduction in switching aggressiveness can momentarily improve THD, although the ANN helps recover consistency by balancing parameter accuracy with harmonic performance.

**Figure 10:**
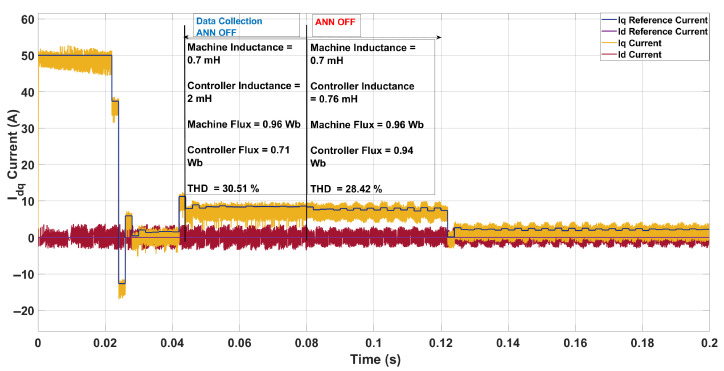
Current in the *dq*-axis for $T_{s}=$ 10 μs with $\lambda_{u}=10$ for a three-step SDA-MPC with Parameter mismatch of L = 0.7 mH and flux = 0.96 Wb.

In the second scenario, shown in Figure 10, the machine inductance was reduced to 0.7 mH while the controller retained its nominal value of 2.0 mH. The flux mismatch was also considerable, with the actual machine flux at 0.96 Wb compared to the controller’s 0.71 Wb. This combined mismatch led to significant distortion in the $I_{q}$ current waveform, particularly visible at the rising edge around 0.042 s, and resulted in an initial THD of 30.51% with an average switching frequency of only 1.17 kHz. After the ANN was activated, it updated the inductance to 0.76 mH and identified the flux at 0.94 Wb. This correction substantially reduced waveform distortion, especially at the falling edge around 0.125 s, lowering the THD to 28.42% and slightly increasing the switching frequency to 1.22 kHz. Although the THD remained higher than the nominal case (13.34%), the improvement illustrates how the ANN mitigates the adverse effects of flux and inductance mismatch, trading a modest increase in switching frequency for enhanced current tracking quality.

**Figure 11:**
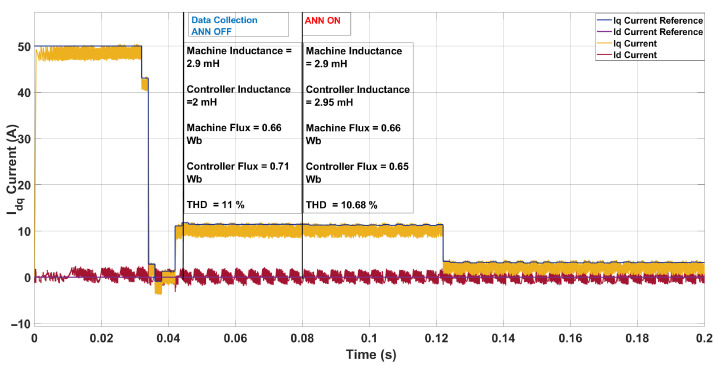
Current in the *dq*-axis for $T_{s}=$ 10 μs with $\lambda_{u}=10$ for a three-step SDA-MPC with Parameter mismatch of L = 2.9 mH and flux = 0.66 Wb.

In the third scenario, shown in Figure 11, the machine inductance was increased to 2.9 mH while the controller retained its nominal value of 2.0 mH. The machine flux was 0.66 Wb compared to the controller’s 0.71 Wb, introducing a moderate mismatch. Prior to ANN activation, this mismatch resulted in a THD of 11.00% with an average switching frequency of 4.25 kHz, already slightly lower than the nominal condition (13.34%). Once the ANN estimator was engaged, it predicted the inductance at 2.95 mH and the flux at 0.65 Wb, both close to their true values. This correction further improved the current waveform quality, reducing THD to 10.68% while raising the switching frequency to 4.73 kHz. The observation that THD is lower than in the nominal case can be explained by the higher machine inductance (2.9 mH versus the nominal 2.0 mH), which acts as a stronger filter on the current ripple. This increased filtering effect suppresses high-frequency harmonics, thereby reducing the measured THD at the expense of slower current dynamics. This scenario highlights the ANN’s ability to refine parameter estimates and enhance current waveform quality, even when the nominal controller already performs relatively well.

When the machine inductance is higher than the nominal inductance used in the controller, the machine naturally provides stronger filtering of the applied voltage ripple. This attenuates high-frequency components in the phase current, leading to a smoother current waveform and therefore a lower THD. The trade-off, however, is that the system becomes less responsive, as higher inductance slows the current dynamics. By contrast, when the machine inductance is lower than the nominal value, the effective filtering is weaker. High-frequency components pass more directly into the current, making the waveform more distorted and leading to a higher THD. In this case, the controller struggles more to enforce smooth current tracking, particularly at fast reference changes. Across the three scenarios, the impact of inductance mismatch on the phase current waveform and THD is evident. When the actual machine inductance exceeds the nominal value assumed by the controller, the higher inductance naturally provides stronger filtering of high-frequency current ripple, yielding smoother waveforms and lower THD. However, this also reduces the current loop bandwidth, causing the $i_{q}$ and $i_{d}$ currents to respond more slowly to reference steps, which is observed as a flattened or delayed rising edge. Conversely, when the machine inductance is lower than the controller’s nominal value, the reduced filtering allows high-frequency components to pass more freely, producing sharper but distorted rising edges and increased THD. Thus, the THD trends and waveform shapes in all scenarios can be understood as the combined effect of natural inductance filtering and the mismatch relative to the controller model.

A summary of the three scenarios, including mismatch values, ANN predictions, and resulting THD values, is presented in Table 3.

### 5.2. System Configuration 2

**Figure 12:**
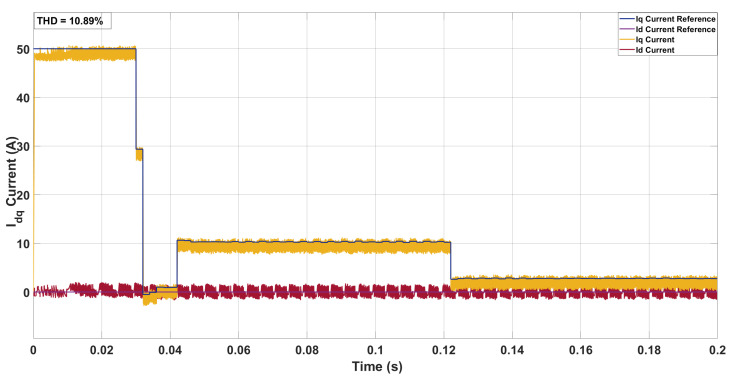
Current in the *dq*-axis for $T_{s}=$ 10 μs with weighing factor $\lambda_{u}=5$ for a two-step SDA-MPC with no mismatch.

Further tests were conducted by retraining the ANN under a two-step prediction horizon with a control effort penalty of $\lambda_{u}=5$. This configuration was applied across the same three mismatch scenarios to evaluate consistency against the three-step results. Under nominal operating conditions (Figure 12), the two-step SDA-MPC produced a phase-current THD of 10.89% with an average switching frequency of 8.02 kHz. Compared to the three-step case, the THD was slightly lower while the switching frequency was noticeably higher, reflecting the reduced penalty on control effort. The higher switching activity enabled more accurate current tracking, consistent with the ANN’s retraining under the two-step cost formulation.

**Figure 13:**
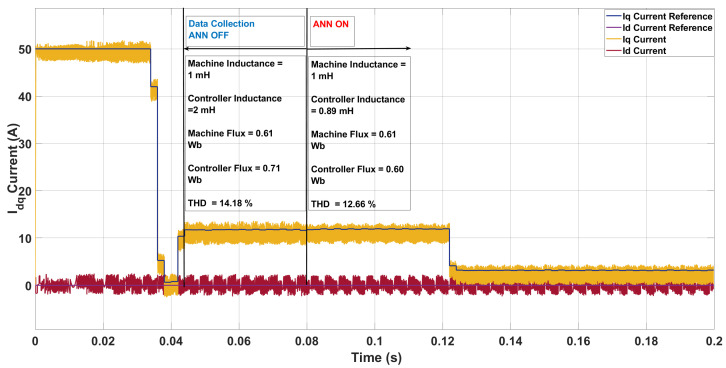
Current in the *dq*-axis for $T_{s}=$ 10 μs with $\lambda_{u}=5$ for a two-step SDA-MPC with Parameter mismatch of L = 1 mH and flux = 0.61 Wb.

In the first case (Figure 13), the machine inductance was reduced to 1.0mH and the flux linkage to 0.61Wb, while the controller retained its nominal values of 2.0mH and 0.71Wb. This mismatch caused pronounced ripple in the $i_{q}$-current tracking and a THD of 14.82% (with a switching frequency of 10.7kHz), compared to the nominal case (10.98%, 8.02kHz). After ANN correction, the inductance was updated to 0.89mH and flux to 0.60Wb, reducing THD to 12.66% and increasing the switching frequency to 12.4kHz. The improvement confirms that the ANN effectively compensated moderate deviations by aligning controller parameters with the plant, though the inherently low inductance limited the achievable THD reduction due to weaker natural filtering.

**Figure 14:**
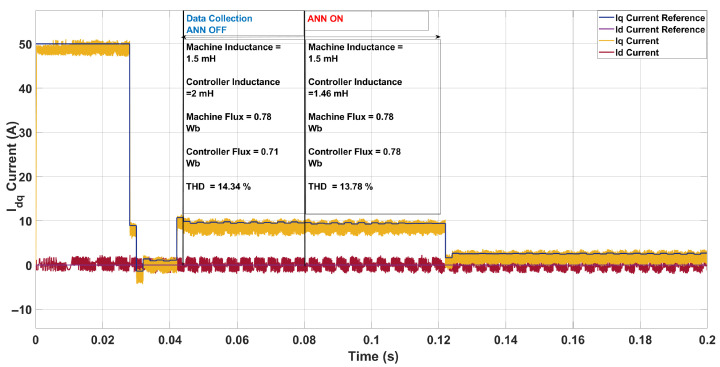
Current in the *dq*-axis for $T_{s}=$ 10 μs with $\lambda_{u}=5$ for a two-step SDA-MPC with Parameter Mismatch of of L = 1.5 mH and flux = 0.78 Wb.

In the second case (Figure 14), the machine inductance was reduced to 1.5mH and the flux linkage increased to 0.78Wb, while the controller retained its nominal settings of 2.0mH and 0.71Wb. This mismatch introduced visible distortion in the $i_{q}$-current tracking, particularly during transient edges, and led to a higher THD of 14.34% compared to the nominal case (10.98%), albeit with a higher switching frequency of 9.7kHz. After ANN estimation, the inductance was updated to 1.46mH and the flux to 0.78Wb. This correction reduced the THD to 13.78% while slightly increasing the switching frequency to 10.0kHz. The improvement, though modest, indicates that the ANN effectively compensated part of the mismatch by restoring more consistent current dynamics. Nevertheless, the THD remained above the nominal value because the lower machine inductance inherently provides weaker filtering of current ripple, allowing more harmonic components to persist in the phase current.

**Figure 15:**
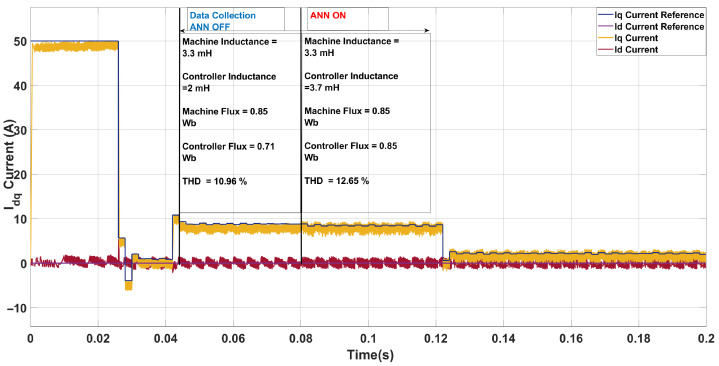
Current in the *dq*-axis for $T_{s}=$ 10 μs with $\lambda_{u}=5$ for a two-step SDA-MPC with Parameter mismatch of L = 3.3 mH and flux = 0.85 Wb.

In the third case (Figure 15), the machine inductance was increased to 3.3mH while the controller continued to operate with the nominal value of 2.0mH. The machine flux was 0.85Wb, and the ANN produced an overestimated inductance of 3.7mH while accurately identifying the flux. As a result, the phase-current THD increased from 10.96% (baseline) to 12.65%, accompanied by a slight reduction in average switching frequency from 6.0kHz to 5.8kHz. This degradation arises because the ANN’s overestimation makes the controller assume a slower current response than the actual plant dynamics. Consequently, the controller applies less aggressive voltage transitions, which weakens current tracking and introduces phase and amplitude errors. In the two-step framework, these errors accumulate over successive horizons, prompting additional but poorly timed corrective actions. The resulting waveform distortion increases harmonic content, outweighing the natural filtering benefit of the higher inductance. Hence, under this condition, the ANN compensation proved less effective, as the inductance overestimation combined with the two-step cost structure to yield a net increase in THD.

**Figure 16:**
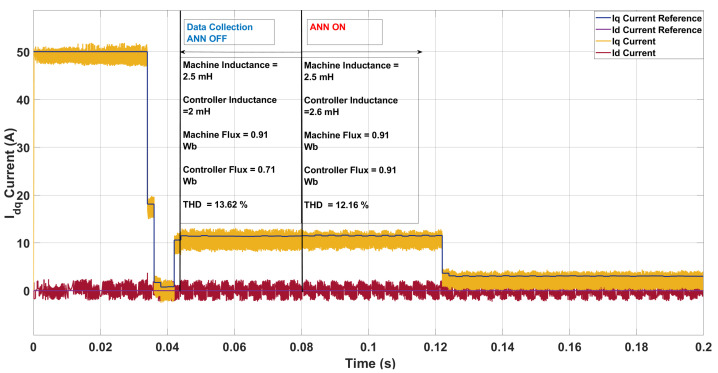
Current in the *dq*-axis for $T_{s}=$ 10 μs with $\lambda_{u}=5$ for a two-step SDA-MPC with Parameter Mismatch of of L = 2.5 mH and flux = 0.91 Wb.

In the fourth case, the machine inductance and flux were 2.5 mH and 0.91 Wb, respectively, while the controller assumed 2.0 mH and 0.71 Wb. As shown in Figure 16, the ANN updated the controller parameters to 2.6 mH and 0.91 Wb, closely matching the true machine values. This correction reduced the THD from 13.62% (with an average switching frequency of 6.8 kHz) to 12.16% (7.1 kHz). The improvement arises because the larger machine inductance provided additional current ripple filtering, while the ANN update compensated for the flux mismatch. Together, these effects smoothed the current waveform and reduced high-frequency harmonics, explaining the lower THD compared to the uncompensated case. In summary, both the two-step and three-step SDA-MPC cases reveal the same underlying principle: when the machine inductance is higher than the controller’s nominal value, natural filtering reduces current ripple and THD, whereas lower inductance weakens filtering and increases distortion. The key difference lies in horizon length. With a longer, three-step horizon, the controller has more foresight to compensate for mismatch, so THD improvements from ANN correction are more pronounced. By contrast, the two-step horizon is inherently more sensitive to parameter errors, meaning that although the ANN still provides benefit, the resulting THD remains higher than in the nominal case. This explains why some mismatch scenarios appear to reduce THD in the three-step case but not in the two-step case—the difference arises from the predictive horizon rather than a contradiction in the filtering effect.

A summary of the cases, its prediction mismatch value, THD and results is summarised in Table 4.

**Figure 17:**
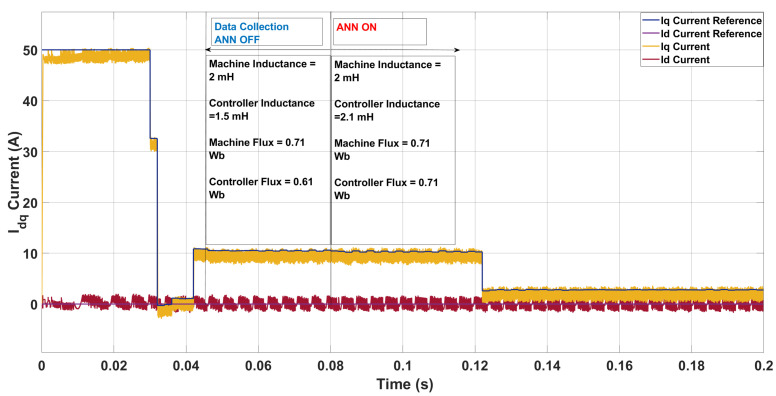
Current in the *dq*-axis for $T_{s}=$ 10 μs with $\lambda_{u}=5$ for a two-step SDA-MPC.

A test was performed in Figure 17 to assess the ANN’s capability to revert controller parameters when the machine returned to nominal settings. Starting with controller parameters being lower than that of the machine with an inductance and flux of 1.5 mH inductance and 0.61 Wb while the machine had the nominal inductance and flux of 2.0 mH and 0.71 Wb respectively, the ANN updated the controller values to 2.1 mH and 0.71 Wb, realigning them with the machine parameters.

**Figure 18:**
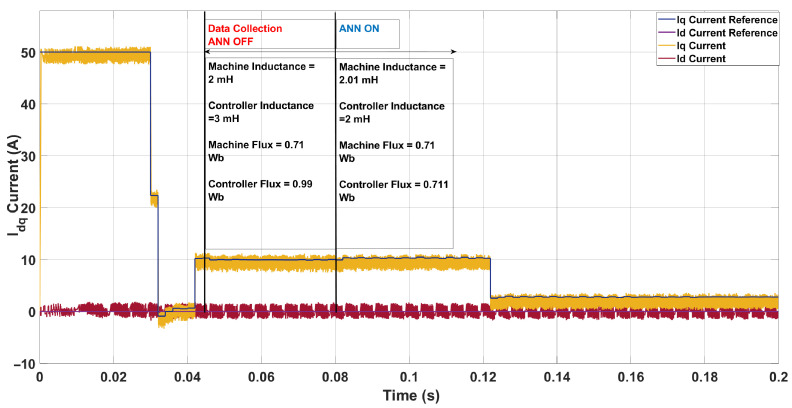
Current in the *dq*-axis for $T_{s}=$ 10 μs with $\lambda_{u}=5$ for a two-step SDA-MPC.

Another test was performed in Figure 18 to assess the ANN’s capability to revert controller parameters when the machine returned to nominal settings. With the controller parameters higher than the machine nominal values. The controller parameter were set at higher values of 3 mH and 0.99 Wb, while the ANN updated them to 2.01 mH and 0.711 Wb, respectively

### 5.3. Lyapunov Stability Analysis

**Figure 19:**
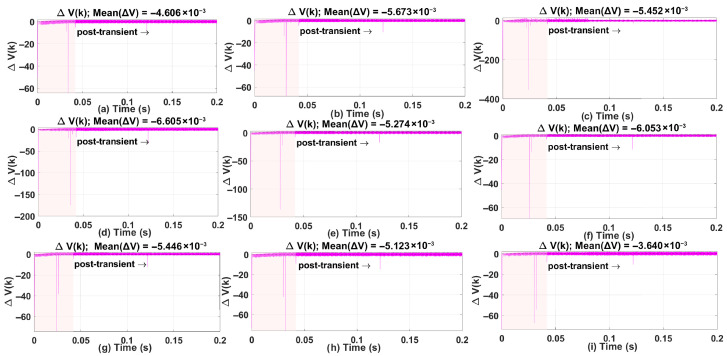
Lyapunov Stability Analysis for ANN-SDA Mismatch Scenerios.

Figure 19 shows nine subfigures of the Lyapunov function V(k) and its difference ΔV(k) for different operating conditions in Section 5.1 and Section 5.2. Each subplot highlights two regions:

- Transient Region (0–0.042 s, shaded pink): This interval contains large spikes in ΔV(k) caused by rapid changes in the tracking error during the initial response. These spikes occur because the controller is compensating for a reference change and the *q*-axis current dynamics dominate this phase, as seen in the current plots in Section 5.1 and Section 5.2. ANN adaptation also begins in this period, contributing to short bursts in ΔV(k).
- Post-Transient Region (after 0.042 s): Beyond the transient, ΔV(k) remains close to zero with a slight negative bias, indicating that the Lyapunov function is bounded and exhibits a non-increasing trend. This region reflects steady-state behavior before and after ANN adaptation stabilizes. For stability assessment, the mean of ΔV(k) is computed in this post-transient window, and the values reported in each subplot confirm practical stability.

Across all scenarios, the Lyapunov function decreases sharply during the transient and remains flat thereafter, while ΔV(k) shows only small oscillations around zero in steady state. These observations confirm that the proposed controller maintains closed-loop stability once the transient phase ends.

## 6. Conclusions

The results clearly demonstrate that ANN-based estimation is an effective real-time compensation strategy for parameter mismatches in PMSM drives. While inductance estimation was not always exact, the ANN consistently provided close approximations, leading to reductions in THD and improved dynamic response. The most notable improvements were observed in cases of flux mismatch, where ANN corrections successfully eliminated waveform deviations and stabilized the $i_{q}$ current trajectory. In contrast, scenarios involving inductance mismatch showed more nuanced behavior: when the machine inductance was higher than the nominal controller value, natural filtering reduced THD even before ANN correction, whereas in lower-inductance cases, the ANN was less effective in suppressing distortion. This highlights that the method is most robust for flux mismatch and moderate inductance deviations, but less so in strongly under-inductive regimes.

A key distinction between the two-step and three-step SDA-MPC schemes lies in their horizon length. The three-step horizon provides greater foresight, allowing the controller to compensate more effectively for mismatch and thereby recover consistent THD levels after ANN correction. By contrast, the two-step case is inherently more sensitive to parameter errors, which explains why THD occasionally increased despite ANN intervention. Thus, the difference is not contradictory, but rather a reflection of the trade-off between prediction horizon length, computational burden, and sensitivity to parameter mismatch.

Nevertheless, some limitations should be acknowledged. The ANN was trained entirely on simulation data, meaning that experimental variability, inverter nonlinearities, and measurement noise were not yet considered study also that the parameter mismatch is introduced instantaneously. Moreover, compensation under low-inductance conditions remains a challenge, as the weaker natural filtering amplifies harmonic content that the ANN cannot fully suppress.

These issues motivate future work, which will focus on experimental validation of the ANN-based predictive control framework under practical conditions, including investigating scenarios where motor parameters vary slowly over time—such as inductance doubling over several seconds—to better reflect practical operating conditions while also observing the independent impact of each mismatch on the current dynamics. In addition, hybrid approaches—such as integrating observers (e.g., ESO or MHE) with the ANN or combining with model-free predictive control methods like recursive least squares—will be explored to improve robustness in problematic regimes. Such extensions will be particularly relevant for aerospace and more-electric aircraft applications, where reliability under diverse operating conditions is critical.

## Figures and Tables

**Table 1:** System model equations for the PMSM drive.

**State Variables**	$x_{k}={\left[\begin{array}{cc} i_{q} & i_{d} \end{array}\right]}^{T}$
**Input**	$u_{k}=S_{\mathrm{abc},k}$
**Output**	$y_{k}={\left[\begin{array}{cc} i_{q} & i_{d} \end{array}\right]}^{T}$
**State Matrices**	$A=I_{2}+A_{xy}\cdot T_{s}$, $B=B_{xy}\cdot T_{s}$, $\psi =\psi_{xy}\cdot T_{s}$, $C=C_{xy}\cdot T_{s}$ $A_{xy}=\left[\begin{array}{cc} -\frac{R_{s}}{L_{d}} & \omega_{e}\left(k\right)\\ -\omega_{e}\left(k\right) & -\frac{R_{s}}{L_{q}} \end{array}\right]$ $B_{xy}=\left[\begin{array}{cc} \frac{1}{L_{d}} & 0\\ 0 & \frac{1}{L_{q}} \end{array}\right]T_{\mathrm{abc}}^{dq}V_{dc}$ $\psi_{xy}=\left[\begin{array}{c} 0\\ \frac{\psi_{m}\omega_{e}\left(k\right)}{L_{q}} \end{array}\right]$ $C_{xy}=\left[\begin{array}{cc} 0 & 1\\ 1 & 0 \end{array}\right]$

**Table 2:** Execution time statistics for SDA-MPC and ANN modules (μs).

Module	Mean	Std	Min	95th-Percentile
SDA-MPC	52.59	39.78	45.03	59.96
ANN Estimator	1.5	0.18	1.26	1.87

**Table 3:** ANN-based parameter compensation under inductance mismatch.

Scenario	Param.	Controller	Machine	ANN Pred.	Pred. Mis. (%)	THD (%)
1	*L* [mH]	2.00	3.30	3.20	3.03	12.54 → 12.11
	ψ [Wb]	0.71	0.76	0.76	0.00	
2	*L* [mH]	2.00	0.70	0.76	8.57	30.51 → 28.42
	ψ [Wb]	0.71	0.96	0.94	2.08	
3	*L* [mH]	2.00	2.90	2.95	1.72	11.00 → 10.68
	ψ [Wb]	0.71	0.66	0.65	1.52	

**Table 4:** ANN-based compensation under parameter mismatch.

Scenario	Param.	Controller	Machine	ANN Pred.	Pred. Mis. (%)	THD (%)
1	*L* [mH]	2.00	1.00	0.94	6.00	14.12 → 12.94
	ψ [Wb]	0.71	0.61	0.62	1.64	
2	*L* [mH]	2.00	3.30	3.70	12.12	10.96 → 12.65
	ψ [Wb]	0.71	0.85	0.85	0.00	
3	*L* [mH]	2.00	1.50	1.46	2.67	14.34 → 13.78
	ψ [Wb]	0.71	0.78	0.78	0.00	
4	*L* [mH]	2.00	2.50	2.60	4.00	13.62 → 12.16
	ψ [Wb]	0.71	0.91	0.91	0.00	

## Data Availability

The raw data supporting the conclusions of this article will be made available by the authors upon reasonable request.
